# Cognitive Manic Symptoms in Bipolar Disorder Associated with Polymorphisms in the *DAOA* and *COMT* Genes

**DOI:** 10.1371/journal.pone.0067450

**Published:** 2013-07-05

**Authors:** Dzana Sudic Hukic, Louise Frisén, Lena Backlund, Catharina Lavebratt, Mikael Landén, Lil Träskman-Bendz, Gunnar Edman, Martin Schalling, Urban Ösby

**Affiliations:** 1 Neurogenetics Unit, Department of Molecular Medicine and Surgery, Karolinska Institutet, Stockholm, Sweden; 2 Center for Molecular Medicine, Karolinska University Hospital, Stockholm, Sweden; 3 Department of Psychiatry, Tiohundra AB, Norrtälje, Sweden; 4 Department of Clinical Neuroscience, Karolinska Institutet, Stockholm, Sweden; 5 Department of Neuroscience and Psychology, The Sahlgrenska Academy at Gothenburg University, Sweden; 6 Department of Clinical Sciences, University Hospital, Lund, Sweden; 7 Department of Neurobiology, Care Sciences, and Society, Centre for Family Medicine (CeFam), Karolinska Institutet, Stockholm, Sweden; University of Illinois at Chicago, United States of America

## Abstract

**Introduction:**

Bipolar disorder is characterized by severe mood symptoms including major depressive and manic episodes. During manic episodes, many patients show cognitive dysfunction. Dopamine and glutamate are important for cognitive processing, thus the *COMT* and *DAOA* genes that modulate the expression of these neurotransmitters are of interest for studies of cognitive function.

**Methodology:**

Focusing on the most severe episode of mania, a factor was found with the combined symptoms of *talkativeness, distractibility,* and *thought disorder,* considered a cognitive manic symptoms (CMS) factor. 488 patients were genotyped, out of which 373 (76%) had talkativeness, 269 (55%) distractibility, and 372 (76%) thought disorder. 215 (44%) patients were positive for all three symptoms, thus showing CMS (Table 1). As population controls, 1,044 anonymous blood donors (ABD) were used. Case-case and case-control design models were used to investigate genetic associations between cognitive manic symptoms in bipolar 1 disorder and SNPs in the *COMT* and *DAOA* genes.

**Results:**

The finding of this study was that cognitive manic symptoms in patients with bipolar 1 disorder was associated with genetic variants in the *DAOA* and *COMT* genes. Nominal association for *DAOA* SNPs and *COMT* SNPs to cognitive symptoms factor in bipolar 1 disorder was found in both allelic (Table 2) and haplotypic (Table 3) analyses. Genotypic association analyses also supported our findings. However, only one association, when CMS patients were compared to ABD controls, survived correction for multiple testing by max (T) permutation. Data also suggested interaction between SNPs rs2391191 in *DAOA* and rs5993883 in *COMT* in the case-control model.

**Conclusion:**

Identifying genes associated with cognitive functioning has clinical implications for assessment of prognosis and progression. Our finding are consistent with other studies showing genetic associations between the *COMT* and *DAOA* genes and impaired cognition both in psychiatric disorders and in the general population.

**Table 1:** Clinical characteristics of the Bipolar 1 patient sample.

Bipolar disorder type 1 [n]	488
Men [n (%)]	209 (43)
Talkativeness [n (%)]	373 (76)
Distracibility [n (%)]	269 (55)
Thought disorder [n (%)]	372 (76)
Cognitive manic symptoms* [n (%)]	215 (44)
Men [n (%)]	81 (39)
Non-Cognitive manic symptoms [n (%)]	248 (51)
Men [n (%)]	117 (56)
Unknown [n (%)]	25 (5)
Men [n (%)]	11 (44)
Anonymous blood donors (ABD)	1044
Men [n (%)]	616 (59)

* having all three symptoms: talkativeness, distractibility, and tought disorder.

**Table 2:** Allelic association in bipolar 1 patients with cognitive manic symptoms (CMS) patients compared to non-CMS and to ABD controls in the *DAOA* and *COMT* genes.

		BP1 CMS	BP1 non-CMS	ABD	BP1 CMS vs. non-CMS^b^				BP1 CMS vs. ABD controls^b^			
Gene	SNP^a^	aa/ab/bb	aa/ab/bb	aa/ab/bb	p	EMP1^c^	EMP2^d^	OR [95% CI] e	p	EMP1^c^	EMP2^d^	OR [95% CI] e
*DAOA*	rs3916967 (C/T)	32/88/89	50/118/77	177/494/361	0.018	0.018	0.21	0.72 [0.55–0.93]	0.029	0.026	0.28	0.78 [0.66–1.0]
*DAOA*	rs2391191 (A/C)	28/75/79	39/111/70	179/487/357	0.055	0.039	0.50	0.75 [0.57–1.0]	0.020	0.019	0.21	0.75 [0.63–1.0]
*DAOA*	rs1935062 (C/A)	26/67/89	35/102/86	146/460/405	0.12	0.12	0.78	0.80 [0.58–1.0]	0.069	0.066	0.52	0.80 [0.65–1.0]
*COMT*	rs5993883 (T/G)	33/120/53	71/112/57	269/510/223	0.025	0.030	0.27	0.73 [0.56–0.95]	0.0017*	1.0E^−4^ *	0.021*	0.68 [0.91–1.4]
*COMT*	rs165599 (G/A)	29/94/87	25/93/126	87/443/501	0.093	0.094	0.69	1.27 [1.0–1.8]	0.014	0.017	0.16	1.34 [1.1–1.7]

a SNP (minor allele(a)/major allele(b)).

b gender and rs1718119 as covariate.

c point-wise p-value from 10,000 pemutations with no covarite (EMP1).

d corrected empirical p-value by max (T) permutation.

e odds ratio (OR), the proportion of minor versus major allele affected (cognitive manic symptoms factor)/proportion of minor versus major allele unaffected (non-cognitive manic symptoms factor or ABD controls).

* significant after correction for multiple testing by max (T) permutation.

**Table 3:** Haplotype association of haplotype group 1 in bipolar 1 patients with cognitive manic symptoms (CMS) compared with non-CMS patients or ABD controls in the *DAOA* gene.

				CMS vs non-CMS^b^			CMS vs ABD^b^		
*DAOA*	rs3916967	rs2391191	rs1935062	F^a^	p	OR [95% CI]^c^	F^a^	p	OR [95% CI]^c^
Haplotype 1	C	A	C	0.32	0.25	0.83 [0.66–1.1]	0.33	0.14	0.83 [0.71–1.1]
Haplotype 2	T	G	C	0.032	0.34	0.64 [0.32–1.1]	0.037	0.19	0.58 [0.37–1.1]
Haplotype 3	C	A	A	0.074	0.077	0.58 [0.39–0.89]	0.075	0.10	0.65 [0.47–1.0]
Haplotype 4	T	G	A	0.57	0.029	1.38 [1.17–1.8]	0.56	0.0057	1.41 [1.1–1.6]

a frequency (F) in sample.

b gender and rs1718119 as covariates.

c odds ratios (OR) for each haplotype.

## Introduction

Bipolar disorder is characterized by severe mood symptoms including major depressive and manic episodes. Mania is a state of abnormally elevated mood and a defining criteria for bipolar 1 disorder diagnosis. During the manic episodes, many patients show cognitive dysfunction. However, there is also evidence for cognitive dysfunction between active mood episodes, contributing to reduced social function among a substantial number of bipolar patients. Cognitive dysfunction has in recent times been emphasized as an important factor in the reduced long term social function of patients with bipolar disorder and also other psychiatric patients, and has been the focus of intensified study.

Since the dopamine (DA) system is essential for normal cognitive performance [1], [2], genes that regulate the dopamine system are of central interest for further investigations of cognitive function. *COMT* is involved in dopamine catabolism, and considered especially important in the prefrontal cortex (PFC) [3]. In a number of studies, *COMT* has been associated with cognitive impairment in several psychiatric disorders, and also in the general population [4]. In several independent studies, D-amino acid oxidase activator (*DAOA*) and Catechol-O-methyltransferase (*COMT*) have been found to be associated with bipolar disorder [5], [6], [7], [8], [9], [10], [11], [12], [13]. The *DAOA* gene acts through the N-methyl-D-aspartate (NMDA) receptors [14] that have a central role in memory function and synaptic plasticity [15] and have been shown to be modified in bipolar disorder [16], [17]. Furthermore, there is support for a gene-gene interaction between *COMT* and *DAOA* in the PFC [18], [19]. The *COMT* and *DAOA* genes may contribute to the pathophysiology of psychiatric disorders, and especially cognitive manic symptoms, by the combined effect of dopaminergic and glutamatergic pathways [18], [19].

Previously, we have shown that a combination of the manic symptoms distractibility (i.e., attention too easily drawn to unimportant or irrelevant external stimuli), talkativeness (more talkative than usual or pressure to keep talking), thought disorder (flight of ideas or subjective experience that thoughts are racing), as defined by the DSM-IV and assessed during manic episodes, form a cognitive manic symptom factor. This factor has been genetically associated with polymorphisms in the *P2RX7* gene in bipolar disorder [20].

The aim of this study was to investigate genetic associations between cognitive manic symptoms during manic episodes in bipolar 1 disorder and SNPs in the *COMT* and *DAOA* genes.

## Materials and Methods

### Ethics Statement

This study was approved by the Regional Ethical Review Board in Stockholm in accordance with the Helsinki Declaration of 1975. The name in Swedish of the ethics committee is: *Regionala etikprövningsnämnden i Stockholm*. In Sweden, ethical committees are separate national government authority. All bipolar participants had full capacity to consent and the informed consent process was both verbal and written during a visit to a special trained psychiatric nurse.

### Participants

Consecutive patients with a clinical bipolar 1 disorder diagnosis, above the age of 18 years, were invited to participate in the study. Patients were recruited from specialized outpatients clinics for affective disorders (n = 373) and regular psychiatric outpatient departments (n = 115) mainly from Karolinska University Hospital Huddinge. Patients were phenotyped with a lifetime assessment of specific symptoms of mania and depression. Focusing on the most severe episode of mania, information was obtained from medical records and also from interviews when necessary. The module for mania in the Schedules for Clinical Assessment in Neuropsychiatry (SCAN) [21] was used to systematically register the DSM-IV manic symptoms: elevated mood, irritability, over-activity, grandiosity, decreased sleep, talkativeness, distractibility, goal-directed behavior, thought disorder, and embarrassing behavior [22]. The factor structure of the manic symptoms was analyzed. A factor was found with the combined symptoms of *talkativeness, distractibility,* and *thought disorder* as previously described [20]. This factor was considered a cognitive manic symptoms (CMS) factor. Thus, all patients fulfilling these three criteria were considered as CMS patients, while the rest of the patients were considered as non-CMS patients. CMS was a categorical, not a quantitative variable. In the present analysis, 488 patients were genotyped, out of which 373 (76%) had talkativeness, 269 (55%) distractibility, and 372 (76%) thought disorder. 215 (44%) patients were positive for all three symptoms, thus showing CMS, while 248 (51%) patients did not and were considered as non-CMS. 25 patients were classified as unknown. As population controls, 1,044 anonymous blood donors (ABD) were used, recruited from Karolinska University Hospital Solna (Table 1).

### DNA Preparation and Genotyping

Venous blood was drawn from each individual. DNA was extracted according to standard procedures. SNPs were selected for genes in the dopamine system, reported to influence risk for major psychosis, using the HapMap database (http://www.hapmap.org). The genotyping process was performed on a 7900HT Fast Real-Time PCR System Instrument using allele-specific Taqman MGB probes labeled with fluorescent dyes FAM and VIC (Applied Biosystems, Foster City, CA, USA), in accordance with the manufacturer’s instructions. Allelic discrimination was performed with the ABI PRISM 7900HT SDS and the SDS 2.2.1 program (Applied Biosystems).

**Figure 1:**
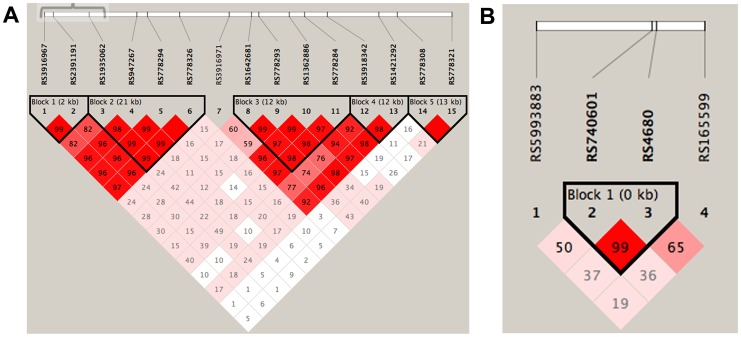
LD structure of a) the *DAOA* gene and b) the *COMT* gene, showing the SNPs analyzed. The numbers in the squares represent the pair-wise Dvalue, empty squares stand for D = 1. Pink-red color indicates a pair-wise LOD >2 with color intensity proportional to D. With squares indicates LOD<2. Haplotype blocks are formed if 95% of comparisions are strong LD that is the 95% CI of Dis within [0.7-0.98]. Haplotype group 1 includes rs3916967, rs2391191, rs1935062.

In the *DAOA* gene, fifteen SNPs (rs3916967, rs2391191, rs1935062, rs947267, rs778294, rs778326, rs3916971, rs1642681, rs778293, rs1362886, rs778284, rs3918342, rs1421292, rs778308, rs778321) were studied (Figure 1a), and in the *COMT* gene, four SNPs (rs5993883, rs740601, rs4680, rs165599) were studied. All SNPs were selected according to earlier published findings. Hardy Weinberg p-value cut-off was p≤0.05 for both cases and controls.

### Statistical Analyses

“Hardy Weinberg equilibrium” was assessed using chi-square tests. In order to investigate genetic associations with cognitive manic symptoms in bipolar mania, SNPs in the *DAOA* and *COMT* genes were analyzed for allelic association in a case-case model, where bipolar patients with cognitive manic symptoms were compared with patients without cognitive manic symptoms, and also in a case-control model, where bipolar patients with cognitive manic symptoms were compared with ABD population controls. In both models, logistic regression with qualitative measurments was used to test for allelic association, where gender and rs1718119 was used as covariates, taking into account our previously published associations with rs1718119 (*P2RX7*) genotypes and cognitive deficit [20]. Associations were corrected for multiple testing by the max(T) permutation in PLINK with 1,000 permutations per SNP. Further, haploblocks were calculated for anonymous blood donors (ABD) using Haploview in PLINK [23]. Haploblocks, including SNPs allele-wise nominally associated to cognitive manic symptoms (p<0.05) or SNPs nearby (D’>0.80), were tested for haplotype distribution difference with χ^2^-test using a sliding window (3 SNP window) approach. Association of specific haplotypes to cognitive manic symptoms was tested using logistic regression with gender and rs1718119 as covariates. A 3 SNP window existed only for *DAOA*. p≤0.05 was regarded significant in the analyses of haplotype distribution. SNPs with nominal allele frequency associated with cognitive manic symptoms (p<0.05) were further tested for genotype association using logistic regression in dominant, recessive and codominant models. An allele by allele epistasis test was performed between the SNPs rs2391191 in *DAOA* and rs5993883 in *COMT* using logistic regression with a multiplicative interaction term, Genotype by genotype epistasis was performed using logistic binary regression with the interaction term rs2391191*rs5993883, where the SNP genotypes were binary categorized according to dominance of the alleles A and T, repectively, using IBM SPSS Statistics version 20.0 (IBM Coporation, USA). All other calculations were performed using PLINK in BC|SNPmax data management and analysis (http://pngu.mgh.harvard.edu/purcell/plink/) [24]. The statistical power to exclude association between CMS and allele frequency of a SNP at alpha = 0.05 was calculated according to http://pngu.mgh.harvard.edu/Bpurcell/gpc/cc2.html.

## Results

All SNPs were in Hardy Weinberg equilibrium. Genotyping success rate was at least 95%. Nominal association for *DAOA* SNPs and *COMT* SNPs to cognitive symptoms factor in bipolar 1 disorder was found in both allelic (Table 2) and haplotypic (Table 3) analyses. Genotypic association analyses also supported our findings. The power to detect allelic association for these SNPs was 0.3–0.5. However, only one association, when CMS patients were compared to ABD controls, survived correction for multiple testing by max (T) permutation.

### DAOA

In the case-case analysis of *DAOA,* the minor alleles of rs3916967, allele C, (OR = 0.72, p = 0.018) and rs2391191, allele A, (OR = 0.75, p = 0.055) were less common among the cognitive manic symptoms patients (Table 2). Thus, allele T in rs3916967 (OR = 1.39, p = 0.018), and allele G in rs2391191 (OR = 1.33, p = 0.055) were nominally associated with cognitive manic symptoms. Likewise, in the case-control analysis, minor alleles of the same SNPs were less common in cognitive manic symptoms patients, rs3916967 allele C (OR = 0.78, p = 0.029) and rs2391191 allele A (OR = 0.75, p = 0.020) (Table 2), thus alleles T (OR = 1.28, p = 0.029) and G (OR = 1.33, p = 0.020) were nominally associated with cognitive manic symptoms. In agreement with the nominal allele frequency association, the leftmost haplotype window (rs3916967, rs2391191 and 1935062) of *DAOA* (Figure 1a) formed a distribution of haplotypes that was different for cognitive manic symptoms patients (χ^2^ = 9.0, df = 3, p = 0.029) compared to both non-cognitive manic symptoms patients and ABD controls (χ^2^ = 6.87, df = 3, p = 0.07). The haplotype consisting of the three major alleles TGA from SNPs rs3916967, rs2391191 and rs1935062, increased the risk for cognitive manic symptoms in both the case-case (OR = 1.38, p = 0.029) and the case-control analysis (OR = 1.34, p = 0.0057) (Table 3). The allele and haplotype associations were further supported by genotypic association tests in the case-case model, rs3916967 (p_dominant_ = 0.016, p_trend_ = 0.018), rs 2391191 (p_dominant_ = 0.020, p_trend_ = 0.055), and rs1935062 (p_dominant_ = 0.042, p_trend_ = 0.11). In the case-controls model, association was found to rs3916967 (p_dominant_ = 0.037, p_trend_ = 0.076), rs 2391191 (p_dominant_ = 0.028, p_trend_ = 0.061), and rs1935062 (p_dominant_ = 0.026, p_trend_ = 0.11).

### COMT

In the case-case analysis of *COMT*, SNP rs5993883 minor allele T (OR = 0.73, p = 0.023) (Table 2) was less common among the cognitive manic symptoms patients and thus allele G (OR = 1.37, p = 0.025) was positively associated with cognitive manic symptoms. In the case-control analysis, both SNPs rs5993883 minor allele G (OR = 1.45, p = 0.0017) (OBS: different minor/major alleles from case-case analysis; major allele T: OR = 0.68) and rs165599 minor allele A (OR = 1.34, p = 0.014) were nominally associated with cognitive manic symptoms (Table 3). The *COMT* haplotype analysis was not analyzed since LD between markers was D’<0.80. None of the other *DAOA* and *COMT* SNPs showed nominal allele frequencies associated (p<0.05) to CMS comparing to non-CMS or ABD. However, the power to exclude true association of the majority of these other SNPs was low (<0.20), whereas rs4680, rs740601 and rs778308 had a power of >0.7.

The interaction term between rs239119 in *DAOA* and rs 5993883 in *COMT* that assesses relationship between cognitive manic symptoms and genotype interactions was statistically significant both in the case-case model (df = 1, Wald = 5.56, p = 0.018, OR = 0.83), and in the case-control model (df = 1, Wald = 94, p = 10^−5^, OR = 0.39). Further, there was a suggestive allelic interaction between rs2391191 in *DAOA* and rs5993883 in *COMT* in the case-control model (df = 1, *X* ^2^ = 5.0, p = 0.025, OR = 0.68).

## Discussion

### Main Findings

The finding of this study was that cognitive manic symptoms in patients with bipolar 1 disorder was associated with genetic variants in the *DAOA* and *COMT* genes, in both case-case and case-control analyses. In *DAOA*, a risk haplotype was associated with cognitive manic symptoms. Furthermore, in the case-control model we could identify interaction between genetic variants in *DAOA* and *COMT*.

### Strengths and Limitations

Bipolar disorder is clinically defined, thus limited knowledge of disease biology and heterogeneity in clinical symptoms are likely to contribute to varying results and to the problems of identification of genetic loci associated with bipolar disorder [25]. There is a genetic overlap with other psychiatric disorders, especially schizophrenia. However, in genetic analyses use of the case-case model helps to reduce the heterogeneity and environmental differences between disease groups [26]. The case-case model may represent a narrow subgroup of bipolar patients, thus more biologically correlated and hence more related to susceptibility genes than bipolar patients in general [27], [28]. In the present study, we primarily applied a case-case design. SNPs with nominal association in the case-case analysis with cognitive manic symptoms were supported by suggestive findings in a haplotype analysis and in a case-control analysis, which could be considered as a semi-replication. The statistical power to exclude true genetic associations for the majority of *DAOA* and *COMT* SNPs showing no association was low.

The patient sample was recruited for bipolar 1 disorder in a population based way, mainly from specialized outpatient clinics for affective disorders (76%). Most of the included patients have had previous hospital treatment. However, since the risk of recurrence with renewed hospitalization in a recent Swedish population study was unevenly distributed among the patients, and smaller than previously anticipated [29], selecting patients only from inpatient care would bias the patient sample towards more severe cases with more frequent hospitalizations. Thus, findings from the present study are likely to be applicable to bipolar 1 disorder patients in general.

A careful phenotyping was performed of the specific symptoms constituting the DSM IV-diagnosis of a manic episode, focusing on the most severe episode of mania. The factor structure of the manic symptoms was analyzed, and talkativeness, distractibility, and thought disorder were found to constitute a cognitive manic symptom factor. We have previously shown this cognitive manic symptom factor to be associated with polymorphisms in the P2RX7 gene in bipolar disorder [20]. Thus, a limitation was that cognitive function was not measured by neuropsychological tests, and there was no information on cognitive function during remission.

### Findings from Other Studies

There is evidence from other studies supporting that cognitive manic symptoms and difficulties in social cognition are present in bipolar disorder patients also during remission or euthymia [30], [31], [32]. Other positive findings using manic symptoms include defining predictors of recurrence from bipolar disorder [21], [33], [34], and genetic associations with *DAOA* including persecutory delusions [21], [33], [34], and juvenile-onset mood disorder [21], [33], [34]. In addition, in bipolar 1 disorder patients it was found that the number of manic episodes correlated to smaller grey matter volume in dorsolateral PFC, [35], which might be a biological correlate of impaired cognition.

### Cognitive Function

Cognitive behavior is processed in the prefrontal cortex (PFC), first studied in schizophrenia patients with stable impairment of cognitive function. In schizophrenia patients the dorsolateral PFC (DLPFC) [36], [37] has been associated with reduced regional cerebral blood flow (rCBF) during neuropsychological testing, reflecting poor DLPFC performance [36], [37]. In addition, equally good results for working memory were found in schizophrenia patients compared to controls, but with increased working memory load, rCBF in the PFC was significantly reduced in schizophrenia patients [38]. Similar low performance levels in adolescents during acute psychosis compared to adult chronic schizophrenia patients were found [39], suggesting cognitive deficit at early stage of the disease and stable cognitive deficit years after disease onset. Further, there is evidence of a correlation between reduced rCBF and a low homovanillic acid (HVA) concentration in cerebrospinal fluid (CSF), suggesting that HVA, the main dopamine metabolite, which have an important role in the dopaminergic pathway [40], also might be of importance for cognitive function.

### COMT and DAOA Function

The dopaminergic pathway is implicated in the neurobiology of cognitive function, and there are genetic associations with functional *COMT* genotypes related to performance on the neuropsychological tests in bipolar disorder, schizophrenia, ADHD, and in the general population [4], [36], [39]. Bipolar 1 disorder patients assessed during depressive and manic episodes showed the methionine (Met) allele of *COMT* to be related to lower test scores compared to healthy controls [4]. The Egan study examined schizophrenic patients, healthy siblings and healthy controls, investigating genetic effects of *COMT* related to neuropsychological test performance. The methionine (Met) variant of Val158Met *COMT* genotype was associated with enhanced cognitive performance and more efficient response in PFC. Furthermore, there was no significant difference between patients, siblings, or controls, thus the *COMT* genotype association with cognitive manic symptoms was found to be independent of psychiatric diagnosis or risk [36]. This finding was replicated by Malhotra in healthy subjects [38]. There are similar findings of cognitive manic symptoms in working memory in schizophrenic patients, healthy siblings, and controls [37]. However, in children diagnosed with ADHD, examined in a family-based control design, the low activity Met allele was associated with reduced performance [39], opposed to the finding in the Egan study. In addition, it has been shown that *COMT* polymorphisms have pleiotropic effect in PFC [41], [42].

The *DAOA* gene acts through the glutamatergic system by activating D-serine, a neuronal signaling molecule, which activates N-methyl-D-aspartate (NMDA) receptors [14]. NMDA receptors have a central role in memory function and synaptic plasticity [15], both shown to be modified in bipolar disorder [16], [17]. There is also evidence that modified NMDA receptors are associated with impaired cognition [43]. In healthy, the variation of *DAOA* polymorphisms affect the concentration of the main dopamine metabolite, the homovanillic acid (HVA), thus affecting dopamine turnover. [44]. This suggests that the modified dopamine regulation may be one mechanism behind the observed associations between *DAOA* and psychiatric phenotypes. In some but not all post mortem schizophrenia samples, the *DAOA* gene has been shown to be over-expressed in the PFC [45], [46].

In *DAOA*, the G allele of rs2391191 in this study associated with cognitive manic symptoms, has previously been associated with worst performance on Quantitative Transmission Disequilibrium test, for allele G homozygotes in a family based study from Finland of individuals ascertained for bipolar disorder [47], suggesting results in the same direction as ours. In addition, schizophrenia patients in a Chinese population [48] and in East Asians [8], [49] were associated with rs2391191, but other studies were not in agreement with these findings [45].

There is evidence of a possible gene-gene interaction between *COMT* and *DAOA* in the PFC. *COMT* and *DAOA* may contribute to the pathophysiology of psychiatric disorders by combining dopaminergic and glutamatergic pathways, and detrimental genotypes of both Val158Met *COMT* (Val allele) and *DAOA* (rs1421292; T allele), suggesting that healthy controls carrying risk genotypes of both *COMT* and *DAOA* would be expected to have less efficient response in PFC [18], [19]. A mice model study found that dopamine receptors regulated NMDA activation via the NMDA receptor R1 subunit (NR1), suggesting that both dopamine and glutamate signaling contribute to neuronal changes [50]. In healthy subjects, the variation of *DAOA* polymorphisms reflect the variation of concentration of the HVA, affecting dopamine turnover [44], thus implicating that the changed dopamine regulation may be one mechanism behind the observed associations between *DAOA* and psychiatric phenotypes.

### Glutamatergic Impairment

Impairment of the glutamatergic system has been implicated in the pathophysiology of both bipolar disorder and schizophrenia [51], [52]. At first psychotic episode, lower glutamate plasma levels was detected in bipolar and schizophrenia patients, indicating impaired glutamate system at an early stage of the illness [16]. In addition to the NMDA receptor family, there are two other families of glutamate receptors, α-amino-3-hydroxy-5-methyl-4-isoxazolepropionic acid (AMPA) and G-protein coupled receptors [14]. Ketamine, a noncompetitive antagonist of the NMDA receptor, induced decrements in free recall, recognition memory and attention in healthy volunteers, suggesting that NMDA receptors may play a direct role in memory [43]. In addition, mRNA expression of glutamate receptor GluR1, a member of AMPA family, was decreased in striatum in bipolar patients [53]. High affinity glutamate transporters, excitatory amino acid transporters 3 and 4 (EAAT3 and EAAT4), did show decreased expression in the striatum in bipolar patients [54].

### Conclusion

Identifying genes that are associated with cognitive functioning is important to improve assessment of prognosis and progression, in order to identify psychiatric patient subgroups. Our finding that cognitive manic symptoms in patients with bipolar disorder was associated with genetic variants in the *DAOA* and *COMT* genes, in both case-case and case-control analyses, supports that impaired cognitive functioning in general might be associated with the *DAOA* and *COMT* genes. Further analyses should include bipolar patients assessed both during manic episodes and during remission, with structured assessment of cognitive function. Assessment of other psychiatric patients with different diagnoses with the same instrument would make it possible to analyze genetic associations with the *DAOA* and *COMT* genes and general cognitive functioning in psychiatry.
